# Introducing Australia’s clinical care standard for low back pain: A new clinical care standard provides evidence-based guidance to help clinicians deliver best care for people with low back pain

**DOI:** 10.1186/s12998-023-00485-1

**Published:** 2023-06-15

**Authors:** Christopher G. Maher, Aline Archambeau, Rachelle Buchbinder, Simon D. French, Julia Morphet, Michael K. Nicholas, Peter O’Sullivan, Marie Pirotta, Michael J. Yelland, Leo Zeller, Nivene Saad, Elizabeth Marles, Alice L. Bhasale, Christina Lane

**Affiliations:** 1 Sydney Musculoskeletal Health, University of Sydney, Sydney, NSW Australia; 2 Canberra Hospital, Canberra, ACT Australia; 3 School of Public Health and Preventive Medicine, Monash University, Melbourne, VIC Australia; 4 Department of Chiropractic, Macquarie University, Sydney, NSW Australia; 5 School of Nursing and Midwifery, Monash University, Melbourne, VIC Australia; 6 College of Emergency Nursing Australasia, Melbourne, VIC Australia; 7 Pain Management Research Institute, Kolling Institute of Medical Research, University of Sydney, Sydney, NSW Australia; 8 School of Allied Health, Curtin University, Perth, WA Australia; 9 Department of General Practice, University of Melbourne, Melbourne, VIC Australia; 10 School of Medicine, Griffith University, Gold Coast, QLD Australia; 11 Arana Hills Medical Centre, Brisbane, QLD Australia; 12 Metro North Health, Royal Brisbane and Women’s Hospital, Brisbane, QLD Australia; 13 Metro South Health, Princess Alexandra Hospital, Brisbane, QLD Australia; 14 Australian Commission On Safety and Quality in Health Care, Sydney, NSW Australia

In September 2022, the Australian Commission on Safety and Quality in Health Care released its Low Back Pain Clinical Care Standard [1]. The Standard covers the early clinical management of people who present with a new acute episode of low back pain. Work on the Standard began in 2020 with a comprehensive review of international and local guidelines. Guided by this evidence, the standard was drafted over a series of meetings by a topic working group comprising representatives of the professions who manage low back pain, NPS MedicineWise, consumer advocacy groups, independent experts, and the Commission. The Standard was sent to professional associations for feedback and the final version was endorsed by 19 professional associations and supported by an additional two.

Clinical care standards differ from clinical practice guidelines as they focus on key areas of care where the need for quality improvement is greatest rather than providing an exhaustive coverage of all aspects of management. A clinical care standard comprises a small number of quality statements that describe the care patients should be offered by clinicians and health services for their health condition in line with current best evidence. Care standards also usually include quality indicators to allow clinicians and health services to measure how well they are implementing the care recommended in the standard.

## Why we need a clinical care standard for low back pain

In Australia, as in most countries, low back pain is the leading cause of disability burden [2]. It costs the Australian health system $4.8 billion annually [3] and it is the most common health reason forcing middle-aged Australians to retire early [4]. This reduces Australia’s gross domestic product by more than $10.5 billion annually [5] and can cause long term financial hardship for individuals. For example, women forced to retire early due to low back pain reach the age of 65 years with less than 10% of the wealth of their peers who remained in the workforce. [6]

The landmark 2018 *Lancet* low back pain series [7, 8] presented the case that the burden of low back pain was amplified by an epidemic of poor care for this condition. The series argued that while we now have a good understanding of how low back pain should be managed, many people with low back pain continue to receive poor health care, either missing out on recommended care or receiving care that is discouraged [7, 8]. The consistent finding of research investigating usual care delivered for low back pain is that no matter the setting, profession or country, there are numerous opportunities to provide patients with better care. [9]

It is easy to understand why poor care of low back pain might arise. For many professions, low back pain is only briefly covered in training programs. Even for the professions where this is not the case, what they were previously taught is now probably out of date. Our understanding of how to manage low back pain has fundamentally changed over the past few decades. Addressing physical and psychosocial barriers to recovery, providing patient education and advice, and promoting self-management and physical activity are now the core aspects of first line care for low back pain. Imaging, pain medicines, bed rest and surgery are now accepted as playing a limited role in most patients with low back pain [10, 11]. For patients who do not respond to this first line care, or where a risk assessment suggests that they may not respond, provision of physical and psychological therapies that address the barriers to recovery is indicated. [12]

## What needs to change?

To ensure that all Australians receive best practice care for low back pain, that is aligned with the Standard, health services will need to adapt, and clinicians’ behaviour will need to change. The professions who manage low back pain will need to stop or reduce the use of some interventions that were traditionally used, and they might need to take up some new approaches that may be unfamiliar to them. Each profession can still play a valuable role in the management of low back pain. This is likely to require the learning of new skills and styles of work for the professions to work more collaboratively with consumers, as outlined in the care model described in the Low Back Pain Clinical Care Standard.

The Low Back Pain Clinical Care Standard provides an excellent guide for how each of the professions can contribute to better care of low back pain. The Standard points to the key areas of care where the need for quality improvement is greatest, by describing what best practice care should entail. It provides eight quality statements describing best practice for assessment (statements 1–3), management (statements 4–7), and review and referral (statement 8) (Box).

**Table:** 

**Low Back Pain Clinical Care Standard** [1]:** fact sheet for clinicians**
Low back pain refers to pain felt in the lower part of the spine (lumbar spine) localised between the twelfth rib and the inferior buttock crease, which is often accompanied by pain in one or both legs
The Low Back Pain Clinical Care Standard aims to improve the early assessment, management and referral of patients with low back pain, and to improve shared decision making about which tests and treatments are most effective in managing low back pain
It covers the early management of an acute presentation of low back pain that is new, recurrent or an exacerbation of chronic low back pain. However, it does not describe the ongoing management of chronic low back pain
**Quality statement 1: Initial clinical assessment**
The assessment of a patient with a new presentation of low back pain symptoms, with or without leg pain or other neurological symptoms, focuses on screening for specific and/or serious pathology and consideration of psychosocial factors. It includes a targeted history and physical examination with a focused neurological examination when appropriate. Arrangements are made for follow-up based on an evidence-based low back pain pathway
**Quality statement 2: Psychosocial assessment**
Early in each new presentation, a patient with low back pain, with or without leg pain or other neurological symptoms, is screened and assessed for psychosocial factors that may affect their recovery. This includes assessing their understanding of, and concerns about, diagnosis and pain, and the impact of pain on their life. The assessment is repeated at subsequent visits to measure progress
**Quality statement 3: Reserve imaging for suspected serious pathology**
Expectations of imaging and its limited role in diagnosing low back pain are discussed with a patient. Early and appropriate referral for imaging occurs when there are signs or symptoms of specific and/or serious pathology. The likelihood and significance of incidental findings are reported and discussed with the patient
**Quality statement 4: Patient education and advice**
A patient with low back pain is provided with information about their condition and receives targeted advice to increase their understanding and address their concerns and expectations. The potential benefits, risks and costs of medicines and other treatment options are discussed, and the patient is supported to ask questions and share in decisions about their care
**Quality statement 5: Encourage self-management and physical activity**
A patient with low back pain is encouraged to stay active and continue or return to usual activity, including work, as soon as possible. Self-management strategies are discussed, and the patient and clinician develop a plan together that includes practical advice to maximise function, limit the impact of pain and other symptoms on daily life, and address individual needs and preferences
**Quality statement 6: Physical and/or psychological interventions**
A patient with low back pain is offered physical and/or psychological interventions based on their clinical and psychosocial assessment findings, with therapy targeted at overcoming identified barriers to recovery
**Quality statement 7: Judicious use of pain medicines**
A patient is advised that the goal of pain medicines is to enable physical activity, not to eliminate pain. If a medicine is prescribed, it is in accordance with the current Therapeutic Guidelines, with ongoing review of benefit and clear stopping goals. Avoid anticonvulsants, benzodiazepines and antidepressants, because their risks often outweigh potential benefits and there is evidence of limited effectiveness. Consider opioid analgesics only in carefully selected patients, at the lowest dose for the shortest duration possible
**Quality statement 8: Review and referral**
A patient with persisting or worsening symptoms, signs or function is reassessed at an early stage to determine the barriers to improvement. Referral for a multidisciplinary approach is considered. Specialist medical or surgical review is indicated for severe or progressive back or leg pain unresponsive to other therapy, progressive neurological deficits, or other signs of serious and/or specific pathology

*Note:* Quick guides for general practitioners, emergency departments and physiotherapists are available at https://www.safetyandquality.gov.au/standards/clinical-care-standards/low-back-pain-clinical-care-standard/information-clinicians

The Standard advocates that imaging should be reserved for patients with suspected serious pathology, as routine imaging does not improve patient outcomes [13]. The clinical assessment should be used to identify those with greater suspicion of serious pathology who require imaging. Patients should be assessed for psychosocial factors that may delay recovery and, if present, these psychosocial factors should be addressed in management. This may be guided by risk assessment tools such as the Orebro Musculoskeletal Pain Screening Questionnaire [14] or STaRT Back Screening Tool [15]. The patient should be provided with education and advice about their condition, with active self-management strategies and physical activity encouraged. The Standard advises that pain medicines should be used judiciously to support continuation of usual activities and recommends avoiding anticonvulsants, benzodiazepines and antidepressants altogether. The advice about opioid analgesics is that they should only be used in carefully selected patients, at the lowest dose for the shortest duration possible. Evidence-based physical and psychological interventions can be offered if self-management and first line care have failed or if the risk assessment suggests that this is likely to be the case. Finally, the Standard recommends review and referral for patients who have not recovered as anticipated or have deteriorated clinically.

## Resources to support adoption of the Standard

The Commission has produced a suite of resources to support adoption of the Standard and to allow clinicians and health care services to monitor how well they are implementing the care advocated in the Standard. The main document provides the eight quality statements, the indicators to support local monitoring, and detailed background information on low back pain. In addition, there are resources for consumers (a consumer guide explaining the Standard, self-management information, common questions about low back pain), quick guides for general practitioners and emergency departments, and fact sheets for clinicians and health care services. All these resources are freely available on the Commission’s website [1]. The Commission is also partnering with the Royal Australian College of General Practitioners and the Australian Physiotherapy Association to develop online educational resources.

## Conclusion

The Low Back Pain Clinical Care Standard meets an urgent need to provide a resource to ensure curricula relevant to low back pain are up to date, and a clear roadmap for existing clinicians to deliver best care for people with low back pain. The Standard presents challenges and opportunities for clinicians (both individually and collectively), policymakers and funders to adopt models of care that are aligned with the evidence to ensure the best outcomes for people with low back pain.
